# Atlanta Academy of Medicine

**Published:** 1876-01

**Authors:** R. W. Westmoreland

**Affiliations:** Reporter


### Atlanta Academy of Medicine.

R. W. WESTMORELAND, M.D., REPORTER.

ATLANTA, November 2, 1875.

Academy met, Dr. John M. Johnson, President, in the chair.

Report of cases:

Dr. Armstrong desired to express himself more fully in regard to the subject of opium antidote.

I desire, Mr. President, to call attention to a case of poisoning from strychnine, as I believe, in the person of a gentleman who had been taking one of the nostrums advertised in the newspapers to cure the habit of taking opium. It was said to be Collins' preparation, or the same as he prepares.

My patient had been taking this nostrum for a short time, and not feeling altogether as comfortable as when under full doses of opium, took a much larger quantity than directed, which caused him to feel drowsy and free from all unpleasant sensations. Encouraged by the pleasant feelings of the increased dose, he again repeated the nostrum in a still larger quantity in the morning before breakfast, and was soon after attacked with spasms of the muscles generally. He was taken while at breakfast with his family, and described the spasmodic action of the masseter muscles as being violent. I saw him in a few minutes, and remained sometime. The convulsive action was of short duration. He stated, upon being questioned, that he had taken no medicine, (not thinking the "opium antidote" could be followed by such results,) nor had strychnine been used about the house for any purpose. I saw him again at 1 P.M. He said he was then satisfied that he had taken strychnine, and told me for the first time that he had been taking this nostrum. He informed me that he had sent for the agent of whom he purchased the drug, and he had told him that the preparation contained strychnine, and that too large a dose would produce symptoms of poisoning. I had always been convinced, from the effects of the "opium antidote," that it contained opium or some of its salts, as well as from the knowledge, too, that the only way to break a confirmed opium eater is to gradually diminish the dose. I had also reason to believe that one or more tonics entered into its composition, but did not suppose, until then, that it contained strychnine. That my patient was poisoned by strychnine, I am sure, and that the "opium antidote" contained it, I am almost as well convinced. I will state further, that out of a family of four, he only had spasms of the muscles.

In mentioning this case, I simply desire to call attention to one of the ingredients, as I believe, of the "opium antidote," so-called.

Dr. Battey reported a case of overdose of Collin's opium antidote, in a person who had been in the habit of using opium, and exaggerating the doses used, thereby having the antidote in too large a portion. On account of the narcotism produced, the preparation had to be reduced in strength. He believed that in each bottle a person uses there is a gradual diminution of the amount of opium to the previous bottle, and an increase of the tonic, on the theory that a gradual withdrawal of the opium tends to destroy the desire.

Dr. Taliaferro called attention to the action of bleeding in puerperal convulsions. Called to see a woman who had been having convulsions from 6 A.M. to 3 P.M., having come on a few hours after pains set in. She had had about twenty or thirty fits. Upon copious bleeding the number of fits gradually decreased. The labor seemed to have been arrested, the os dilated sufficiently to introduce the finger. Barnes' dilators and the forceps were used to hasten the delivery, which was soon accomplished, the fœtus being dead. Thirty-six hours after the woman died from what he thinks was uræmic poison.

Dr. John M. Johnson desired to know why he attributed the death to uræmia.

Dr. Taliaferro replied that he considered true eclampsia as synonomous with uræmia—in other words, that eclampsia, as a rule, is the result of uræmic poisoning.

Dr. John M. Johnson remarked that he has always held the opinion that uræmic poisoning was preceded by albuminuria and œdema of limbs. Desired to hear some expressive experience on that subject.

Dr. Battey replied that he had witnessed uræmic poisoning without albuminuria or swelling. Cited a case under treatment at present, in which there is uræmic poison without albuminuria or swelling, yet the urine is scanty, with some indication of convulsions, limited to the pectoral muscles. Full doses of bromide of potassium relieved the uræmic symptoms, with a tendency to diuresis. He is of the opinion that in this case there is some connection between the convulsion, scarcity of urine and some cerebral trouble, the latter symptom being more or less marked, and which was caused by the uræmic poison in the blood. He mentioned another case in which there was œdema of lower limbs, with no albuminuria, and without convulsions. He does not remember to have seen uræmic poisoning from albuminuria in advance of pregnancy. Does not regard the absence of albumen as contraindicating uræmic poisoning.

Dr. Todd inquired of Dr. Taliaferro if there was any attempt on the part of the stomach, in the case reported by him, to get rid of the uræmic poison, or could there be any poison without such attempt.

Dr. Taliaferro—There was no diarrhœa or vomiting, and did not know that there was any such attempt made. His experience lead him to think it was not usual, he having never heard of such an occurrence.

Dr. John M. Johnson, with Dr. Logan, saw a case (Mrs. B.) with convulsions. The lady was delivered eight days previously of a healthy child, everything passing well. On the eighth day, in the morning, on account of some sick stomach, followed by vomiting, soon after, she had an action on her bowels. When she got up from the chamber she was seized with a convulsion. At 11 o'clock P.M. she was pulseless, breathing with difficulty and screaming with pain. This continued for four hours. Shoulders and back were cold, and mustard, hot turpentine and lard were applied, with hot injections into the bowels. Elix. of opium and brandy were given three or four hours before any capillary reaction. The exciting cause of this attack was supposed to be the exposure to a draught of air for a few minutes. The right lung seemed to be the seat of trouble, yet nothing was discovered to warrant such belief. A large blister was applied to the chest and back, with slight return of warmth, but no increase in pulse, with the exception of a spasmodic throb now and then. Two doses of ten grains each of quinia were given, with vomiting on the second. Tried, then, by injection, without effect. In twenty-four hours the lady died, seemingly, of asphyxia. Diagnosis, violent conjestion of thoracic viscera.

Dr. Battey inquired if a post-mortem was had. Did prostration come on after action on bowels?

Dr. John M. Johnson.—No post-mortem was had. The prostration seemed to come on from the vomiting. She took in course of the day ℥ij of McMun's elix. On inquiry as to the existence of internal hemorrhage, he replied that there was no evidence of it.

Dr. Taliaferro thought there was no satisfactory explanation of the sudden collapse but hemorrhage into the peritoneal cavity.

Dr. Battey formed a theory upon the hypo-fibrinous state of the blood consequent upon the puerperal condition. Remarked that disordered condition of the stomach, from indigestion and vomiting, was depressing to the already enervated vital powers, and combined to produce heart-clot, or embolism, in the pulmonary vein, preventing the return of blood.

Dr. John M. Johnson thought at the time that it was attributable to this cause, or that some vessel had ruptured.

Dr. Battey remarked that in heart-clot post-mortem shows a white fibrinous coat filling up the lower cavity of heart, and believed that this would have been demonstrated in this case.

Academy adjourned.

---

Atlanta, Ga., November 9, 1875.

The meeting was called to order, Dr. John M. Johnson, President, in the chair.

Report of cases:

Dr. John M. Johnson referred to the case reported by him at the last meeting, wherein he gave it as his diagnosis that the lady died of emboli, as opposed to that of Dr. Logan's, which was rupture of one of the valves of the heart. Upon mature consideration, he has become satisfied that the latter is preferable, and took the opportunity of expressing this conviction.

Dr. Miller, on the discussion as to the efficacy of bleeding in puerperal convulsions, remarked that as the eclampsia was independent of the brain, it originated in an irritation of the spinal cord, and bleeding was beneficial only as preventing secondary injury. The action of these two nervous centres is separate and distinct, and from that view could not conceive how bleeding benefited the condition; but by retarding or lessening the flow of blood to the brain, which, being stopped in its return through the jugular veins by the spasmodic contraction of the cervical muscles, produced congestion of the brain. Uræmic poisoning creates perverted action of the cord, and, as bleeding is not calculated to lessen the uræmic influence, cannot see the utility in it but in preventing lesions of the brain, ending in effusion. In a case where the convulsion is caused by irritated condition of uterus, he thought the convulsions might be more readily controlled than where they were from uræmic poisoning. His chief reliance is chloroform, both as preventive and cure. Does not depend on opium, because it lessens the influence of the will over the spine.

Dr. Taliaferro contended that with the fact of bleeding preventing congestion and effusion of the brain, it also had a sedative effect upon the spine.

Dr. Miller did not see that there was any sedative effect in the remedy. In tetanus, and kindred conditions of the cord, the spasmodic action seems not to be influenced by the blood-letting.

Dr. Taliaferro thought that bleeding modified the spasmodic or irritated condition of the cord in tetanus. As he believes true eclampsia to be the result of uræmic poisoning, his mode, after getting a powerful sedative influence from the bleeding, is to administer chloroform.

Dr. Miller then stated that his purpose in blood letting was to secure the brain against secondary injury, such as effusion, etc., and chloroform was his remedy for the cure of the eclampsia.

Dr. John M. Johnson thought that blood letting was of the first importance in convulsions. Mentioned the case of a young woman afflicted with convulsions. The attending physicians had given assafœtida by enema. On his suggesting bleeding, no one agreed, but in defiance of all he bled. After a quart of blood was drawn she commenced to recover. Thought chloroform was a great remedy, yet he had followed the plan of bleeding and purging with satisfactory results, never having lost a patient. He is led to the belief that uræmic poisoning is attended with œdema, never having seen a case without it, but that albuminuria is not so invariable an attendant.

Dr. Baird discussed the connection of the brain with the cord in eclampsia. Disagrees with Dr. Miller as to their entire separation. Believes bleeding to be a beneficial depletant to the nervous system in eclampsia, but indiscriminate bleeding is very unscientific, the states of plethora and anemia determining the necessity or utility of the remedy. Thought bromide of potassium, cloroform and opium would be beneficial in benumbing the nervous centres. He is satisfied that uræmia may exist without albuminuria, and that albuminuria may be without uræmia.

Dr. Taliaferro agrees with Dr. Baird as to the foolishness of indiscriminate bleeding. His success has led him to always bleed in eclampsia, if circumstances are favorable. Reported the case of a stout negro woman, who, when he saw her, was having convulsions. She had one convulsion while he bled her. After bleeding stopped she was perfectly conscious, and had no more fits. In several other instances the practice has been successful.

Dr. Miller, on the mortality of eclampsia, remarked that it was not a very serious affection; that in many cases the patient would recover without anything.

Dr. Taliaferro thought that the profession would not sustain Dr. Miller in his assertion.

Dr. John M. Johnson was of the opinion that puerperal convulsions were not to be dreaded to such a great extent. In his practice of bleeding the plethoric and building up the anemic, he has never lost a case.

Dr. Baird inquired of Dr. Johnson if he had not found convulsions to occur as often in anemic as in plethoric subjects.

Dr. John M. Johnson replied that his experience led him to think that plethoric patients were more subject to the attacks. Mentioned the case of a women who was stricken blind just before the birth of a child. At the time of labor she had convulsions. As she was anemic, chloroform was resorted to. Eyesight was restored, and the patient recovered, although much emaciated.

Academy adjourned.

---

ATLANTA, Nov. 16, 1875.

Academy met, Dr. John M. Johnson, President, in the chair.

Report of cases:

Dr. W. F. Westmoreland exhibited two specimens of tumors excised by himself, and reported the case of each. The first was an encephaloid cancer of testicle in a boy aged $3\frac{1}{2}$ years. Eighteen months ago the testicle commenced to enlarge; as hydrocele was supposed to be present, the testicle was tapped, with the escape of a turgid fluid. This fluid, as was afterward proven, was from a cyst, one at each extremity of the testicle.

Dr. Baird asked what was the appearance of scrotum, and if the cancer was hereditary, and if it was not an unusual thing for the boy to be healthy in the presence of cancer.

Dr. W. F. Westmoreland replied that the skin of the scrotum had a natural appearance; the disease was not hereditary. Remarked that the affection had not got to the stage of infecting the system thoroughly. As to the return of the cancer, his experience is varied. In some cases of a very recent origin he has had a return, while again the worst forms have not re-appeared on extirpation.

Second specimen was of a tumor of superior maxillary; supposed non-malignant. This was a perfect specimen of a fibrocystic tumor, resembling somewhat an enchondroma. It was on the left side, hanging over the inferior maxillary, and projecting up under the floor of the orbit. The patient recovered very speedily from the operation, and no bad effects followed.

Dr. John M. Johnson reported two cases of diphtheria. The first, a girl of 11 years, had all the symptoms of the disease, but is now convalescent. Second case, a little girl, with all the symptoms of diphtheria, having been taken sick nine days ago. In a very critical situation. Treated with nitrate silver, chlorate potash, quinine, and nourishment by milk and animal essences. Glycerine was used for moistening the throat.

Dr. W. F. Westmoreland inquired what were the first symptoms.

Dr. Johnson replied, that rigors and fever, with influenza, ushered in the disease.

Dr. W. F. Westmoreland asked if he was of opinion that the disease was contagious.

Dr. Johnson replied that he was not prepared to accept the doctrine, though he generally took precaution to prevent contagion.

Dr. Baird inquired what were the physical signs denoting extension of the diphtheritic membrane into the bronchi.

Dr. Johnson answered that the short, whistling sound, as the air passes through the bronchi, and the smothered-like sigh, denote such a condition.

Dr. W. F. Westmoreland asked if the membrane had been examined by microscope, or if the throat had been examined down to any extent.

Dr. Johnson replied that no microscopic examination had been made. Had seen the throat, and called the attention of the Academy to the importance of keeping the throat moist.

Drs. Westmoreland, Johnson and Baird here entered into discussion as to similarities and dissimilarities of croup and diphtheria. Dr. Johnson contending that a patient may have croup and diphtheria, while Dr. Westmoreland thought that croup and diphtheria were distinct affections.

The discussion was terminated by Dr. Westmoreland giving as his opinion that in diphtheria the membrane contained vegetable organisms, to which Oertel has given the name of micrococci—that these organisms are the means of its propagation, and during the disease are found in the blood; while croup is a simple form of inflammation, in which a fibrous exudation occurs on the mucous membrane. Academy adjourned.

